# Editorial: Minimally invasive surgery as a mean of improving fertility: What do we know so far?

**DOI:** 10.3389/fsurg.2023.1203816

**Published:** 2023-05-15

**Authors:** Antoine Naem, Antonio Simone Laganà

**Affiliations:** ^1^ Faculty of Mathematics and Computer Science, University of Bremen, Bremen, Germany; ^2^ Department of Obstetrics, Gynecology, Gynecologic Oncology, and Senology, Bethesda Hospital Duisburg, Duisburg, Germany; ^3^ Unit of Gynecologic Oncology, ARNAS “Civico – Di Cristina – Benfratelli”, Department of Health Promotion, Mother and Child Care, Internal Medicine and Medical Specialties (PROMISE), University of Palermo, Palermo, Italy

**Keywords:** endometriosis, fibroids and fertility, minimally invasive surgery, infertility, in-vitro fertilisation (IVF)

**Editorial on the Research Topic** Minimally invasive surgery as a mean of improving fertility: what do we know so far?

Infertility is defined as the inability to conceive after one year of regular and unprotected sexual intercourse. However, this timeframe is reduced to 6 months when the female patient's age is 35 years or more (1). The lifetime prevalence of infertility was reported to be 17.5% and the 12-month period prevalence was estimated to be 12.6% (2). According to the World Health Organization, the female factor accounts for 37% of infertility cases. Nonetheless, male and female factors coexist simultaneously in about 35% of cases (3). Infertility carries a heavy burden on the couples' life and may cause serious psychologic disorders. In a recent study, 58% of infertile women reported depression, 24% reported anxiety, and another 24% reported both of them (4). Alternatively, a significantly reduced quality of life was found among infertile male patients (5). Therefore, infertility could be considered as a prevalent and serious health issue that should be accounted for and dealt with efficiently. Although Assisted-Reproduction Technologies (ART) could be considered the cornerstone of the fertility treatments, the concept of “*Reproductive Surgery*” was introduced lately and gained a growing interest in the field of reproductive medicine. The main goal of such interventions is improving the pregnancy and live birth rates, with or without ART. This could be done through adhesiolysis, excising intrapelvic pathologies, and restoring the normal pelvic anatomy. In this research topic, we aimed to gather evidence and feature recent trends in the field of *reproductive surgery*.

One of the earliest and widely accepted implications of *reproductive surgery* is performing salpingectomies to optimize the in-vitro fertilization (IVF) outcomes in women with hydrosalpinges (6). However, the optimal timing of this procedure is not precisely determined yet. In their research paper, Yilei et al. demonstrated that oocytes' retrieval 4–6 and 7–12 months postoperatively yields in higher accumulated pregnancy and live birth rates in comparison with retrieving the oocytes 3 months or less after surgery. This study also addressed the dilemma of which intervention should be carried out first: salpingectomies or oocytes retrieval? Although the clinical pregnancy and live birth rates were comparable among the included sample, patients younger than 35 years receiving salpingectomy first exhibited higher pregnancy and live birth rates than those who had their oocytes retrieval first. A similar but non-significant trend was observed in the live birth rates of patients older than 35 years receiving salpingectomies before oocytes retrieval (Yilei et al.). In a different context, Jiang et al. investigated the efficacy of using testicular sperms instead of ejaculated sperms for intracytoplasmic sperm injection (ICSI) in couples with a previously failed embryo transfer cycle. The authors concluded that testicular sperms result in a higher rate of transferable embryos and lower embryonic fragmentation rate. The higher quality of embryos resulting from testicular sperms was accordingly reflected in a better implantation rate than that of embryos resulting from ejaculated sperms (Jiang et al.).

The role of *reproductive surgery* extends further to include the management of uterine fibroids and endometriosis; two of the most common gynecologic diseases that are known to have negative drawbacks on the female fertility. Uterine fibroids are suggested to interfere with fertility through increased uterine contractions, altered uterine microenvironment (7), and abnormal vascularization (Mercorio et al., 8). Although a definitive relationship could not be drawn yet, fibroids that distorts the endometrial cavity (9), or those with a maximum diameter of at least 3 cm (10), are suspected to correlate with infertility. Therefore, myomectomy was favored in similar situations rather than performing it as a standard procedure to treat any uterine fibroid in patients with subfertility. This recommendation is mainly based on the fact that myomectomies cause increased adhesiogenesis (11), especially those that consist of posterior hysterotomy and/or large uterine incisions (Mercorio et al.). Posterior myomectomies are of special importance in this context as they were found to induce more adhesions that could involve the ovaries and cause subsequent fertility problems (12). In the absence of effective anti-adhesive measures, balancing the risks and benefits of this procedure before performing it is highly recommended (Mercorio et al.).

On the other hand, endometriosis is known to cause infertility in almost 50% of patients (13). A recent meta-analysis concluded that excising deep endometriotic lesions improves IVF outcomes (14). Similarly, cystectomy through traction and counter-traction was considered to be the gold standard for treating ovarian endometriomas (15). However, CO_2_ laser vaporization was recently introduced as at least a safer alternative to cystectomy for the treatment of ovarian endometriomas (Candiani et al.). It functions by vaporizing the endometriotic lining of the pseudo-cyst without removing the fibrous capsule of the endometrioma. This approach is thought to provide comparable results to the traditional cystectomy but also a higher preservation of the ovarian reserve (16). This is mainly achieved by the precise ablation, limited penetration of the laser beam, and minimal lateral heat spread of the CO_2_ laser. Although CO_2_ laser vaporization was found to improve ART outcomes, its effect on natural conception could not be determined (Candiani et al.). On the other hand, the timing of endometrioma treatment is a matter of debate as it is not clear when it is the best time to operate them in patients with infertility (17).

The role of minimally invasive surgery in improving the fertility chances seems promising with very good preliminary results from different studies on different clinical scenarios. However, the results could be generally described as indecisive since it is mainly based on retrospective studies. In the management of infertility, timing is a substantial factor that should be accounted for in future research projects. This especially implies on the endometriosis research where the timing and type of surgery are always debated (18). It is substantial to know how different types and classifications of endometriosis influence the fertility chances, and what would be the optimal management in terms of safety and efficacy. Until then, it is crucial to always keep in mind that regardless of the underlying illness- wisdom lies in knowing when not to operate, especially when the risks out-weight the benefits.
